# Tailoring the Home‐Start program to the needs of first‐time parents: A qualitative study

**DOI:** 10.1002/imhj.70048

**Published:** 2025-09-30

**Authors:** Anne M. E. Bijlsma, Leonie M. de Vries, Romy S. Drent, Aurelie M. C. Lange, Geertjan Overbeek

**Affiliations:** ^1^ Research Institute Child Development and Education Research Group Forensic Child and Youth Care Research Group Preventive Youth Care University of Amsterdam Amsterdam The Netherlands; ^2^ Faculty of Applied Social Sciences and Law Research Group Child and Youth Social Care Amsterdam University of Applied Sciences Amsterdam The Netherlands

**Keywords:** early parenting, first‐time parents, qualitative study, volunteer‐led parenting support

## Abstract

Although first‐time parents have a great need for effective support in the first challenging years of child upbringing, there is little research on volunteer‐led parenting support programs in the context of the transition to parenthood. Therefore, this qualitative study examined the perceived value and key components of Home‐Start, a volunteer‐led parenting support program aimed at parents who have everyday parenting questions and receive little support from their environment, in the transition to parenthood. Semi‐structured interviews were conducted among mothers (*N* = 10) with a first child up to 1.5 years old who were enrolled in the Home‐Start program in the Netherlands, and volunteers (*N* = 12) who supported these parents. Important outcomes of the Home‐Start program were increased positive parent‐child interactions, improved parental confidence, and an expanded social network. Identified core needs of first‐time mothers in the Home‐Start program were social, emotional, informational, and instrumental support needs. Suggestions were provided to better align the program's structure with challenges in the transition to parenthood. For example, facilitating the parent‐volunteer match during pregnancy, and developing a module in the Home‐Start training for volunteers aimed at providing structured and informative support based on the latest insights into infant and postpartum care.

## 1 INTRODUCTION

Family interventions that optimize the development of children may have substantial impact on a variety of health outcomes throughout their lives (Campbell et al., 2014; Kelly, 2018). Unfortunately, child and family services are under pressure worldwide, as shown by high caseloads, understaffing, long waiting times for families, and high turnover rates of child and family social workers (Bijlsma et al., 2022; Burns et al., 2023; Griffiths et al., 2023; Gubbels et al., 2021; Lushin et al., 2023; Nilsen et al., 2023; Radey & Wilke, 2023). This might be especially problematic for first‐time parents, who have a great need for reliable information and effective support in the first few challenging years of child upbringing (Svenson et al., 2006). Even though the early years represent a critical period in a child's development, parents have few opportunities to gain an understanding of what to expect in the first few weeks after birth, and may therefore feel unprepared for the demands of new parenthood (Deave et al., 2008; Entsieh & Kristensson Hallström, 2016; Padgett et al., 2024). Deploying volunteer‐led parenting support programs may be a promising and cost‐effective approach to unburden professional practitioners and to prevent family dysfunction at an early stage (Barnes et al., 2009). Currently, there is little research on volunteer‐led parenting support programs in the context of the transition to parenthood (McLeish & Redshaw, 2015). Therefore, this qualitative study aimed to examine the perceived value and specific components of Home‐Start, a volunteer‐led parenting support program, in the transition to parenthood based on the experiences of first‐time mothers and Home‐Start volunteers.

### 1.1 Volunteer‐**l**ed parenting support programs

Volunteer‐based parenting support can be a valuable and cost‐effective alternative to professionally led interventions, as such programs have been shown to positively impact parental emotional and social well‐being, enhance parenting attitudes and confidence, and, in the long term, reduce child behavior problems (Dowling, 2019; Hermanns et al., 2013; Scavenius et al., 2020). In general, a safe and nurturing relationship between parents and volunteers engenders a genuine experience of belonging for parents, and is therefore one of the main components of success in volunteer‐based home visitation programs (Dowling, 2019; Gross Manos et al., 2023). In addition, support from the community may be more culturally adapted and experienced as less judgmental compared to professional support (Gross Manos et al., 2023). For example, volunteers with the same immigration background and ethnic origin as parents are uniquely positioned to support new mothers in navigating the challenges of both new parenthood and integration into a new society (Cwikel et al., 2017). Further, many volunteer‐led parenting support programs offer the option to match parents with volunteers who speak their preferred language (Hamari et al., 2022). Consequently, peer support offered by local organizations may be especially approachable and helpful for parents in disadvantaged circumstances (e.g., poverty, social isolation, single parent households, or being a recent migrant), as they may experience less knowledge of child rearing, and are less likely to access maternity and child health services(McLeish & Redshaw, 2015; Samdan et al., 2022).

It should be noted, however, that volunteer‐based parenting support cannot always replace professional help, for example when clinical problems arise in families such as postnatal depression in mothers (Barnes et al., 2009). Furthermore, effective volunteer support requires comprehensive guidance and supervision to adequately monitor volunteers and program outcomes for families (Grace et al., 2018). This may be a challenge, as voluntary organizations often operate with limited funding for appointed supervisory roles.

**Key findings**

- Participation in the Home‐Start program in the Netherlands resulted in increased positive parent–child interactions, improved parental confidence, and an expanded social network of first‐time mothers.
- Effective elements of the Home‐Start program for first‐time parents were an informal and equal parent–volunteer relationship, and the way volunteers function as experience experts.
- Home‐Start can be strengthened by better aligning the program's structure with challenges in the transition to parenthood.

**Statement of relevance for infant and early childhood mental health**

Although the transition to parenthood can be a stressful and lonely time for some parents, social support at home or in local communities can be a protective factor in preventing adverse health outcomes for families by reducing parenting stress and improving parental self‐esteem. The results in this study emphasize the value of the volunteer‐led Home‐Start program in supporting vulnerable first‐time parents to prevent family dysfunction at an early stage.

### 1.2 Social support needs for first‐time parents

Pregnancy and the transition to parenthood feature two life changes that may be stressful for many parents (Deave et al., 2008). The transition to parenthood not only generates stressors directly derived from parenting (e.g., role overload or parent‐child relationship conflict), but for some parents also produces new stressors such as financial strain, time constraints, and conflicts between partners and a decline in marital quality (Nomaguchi & Milkie, 2020). Importantly, research shows that such strains may also be related to a heightened risk for children of the development of depression and anxiety in adulthood (Batten et al., 2004; Curley et al., 2011). Although the transition to parenthood can be a stressful and lonely time for some parents, social support at home or in local communities can be a protective factor in preventing adverse health outcomes for families by reducing parenting stress and improving parental self‐esteem (Cohen, 2004; Hanna et al., 2002).

Social support for first‐time parents can be differentiated in terms of three types of functional elements: instrumental support (e.g., financial assistance or practical help with daily tasks), informational support (e.g., advice or guidance), and emotional support (e.g., empathy or reassurance) (Cohen, 2004). Literature indicates that for women specifically, instrumental support (i.e., household chores and infant care) is of significant importance in the postnatal period (Leahy‐Warren et al., 2012). In addition, receiving emotional support from a social network may decrease frustration, anxiety, and isolation, and strengthen emotional well‐being, self‐sufficiency, and self‐esteem among new fathers and mothers (Shorey et al., 2015; Svenson et al., 2006; Warren, 2005). Given that some women may feel overwhelmed and unprepared in the transition to parenthood, informational and emotional support aimed at altering unrealistic maternal expectations may reduce feelings of inadequacy (Nelson, 2002).

Social support can be further distinguished into informal support, provided by family, friends, and relatives, and formal support, offered by professionals such as midwives (Samdan et al., 2022). Besides attending classes for learning how to avoid medical interventions during labor, new mothers also wish to be informed by professionals, obtain advice from them, and discuss parenting issues with both professionals and peers (Svenson et al., 2006). Both types of support are associated with improved maternal self‐efficacy, especially in first‐time mothers (Samdan et al., 2022).

### 1.3 Home‐Start

Home‐Start is a worldwide informal (volunteer‐led) home‐visiting program that aims to address social needs of families under stress in local communities, helping to prevent family crises and breakdown (Frost et al., 2000). Home‐Start focuses on parents who have everyday parenting questions, experience a reduced sense of parental competence, and receive little or no support from their social environment. In the Netherlands, parents can either enroll in the program themselves or be referred directly by professionals, such as general practitioners, social workers, or teachers. Home‐Start coordinators meet with the parents to discuss their needs, and match the parent with a local volunteer under their supervision. The volunteer, who also has personal parenting experience, visits the family weekly, for an average duration of one year. There is a mandatory training for new volunteers, consisting of six short e‐learning modules and six half‐day in‐person training sessions. The volunteer encourages parents to establish and strengthen connections with others and helps them learn to utilize local community resources (Meijer & Hollander March, 2019). Home‐Start volunteers aim to enhance parental sense of competence through practical assistance and emotional support (Deković et al., 2010). This Home‐Start approach is grounded in Bandura's theory of self‐efficacy: when parents believe they are capable of positive parenting, and that their actions will positively influence their children's behavior, parents are more likely to demonstrate positive parenting skills (Bandura, 1997; Deković et al., 2010).

In populations of caregivers with children 1.5 years and older, Home‐Start has been shown to effectively improve parenting behavior and parental self‐esteem, and reduce disruptive child behavior through increases in parental self‐efficacy (Asscher et al., 2008; Deković et al., 2010). In a subsequent study, those effects were proven to be as equally effective as other evidence‐based parenting programs (e.g., Video Home Training) (Smallegange et al., 2018). To date, little is known about which components of the Home‐Start program, or volunteer‐led home visiting programs in general, are most effective. Identifying the elements that contribute to desired program outcomes could improve the efficiency, and potentially the effectiveness of Home‐Start, by guiding decisions about which components should be prioritized during program delivery (Leijten et al., 2021). A recent explorative study based on interviews with Home‐Start parents with preschool‐ or older children and volunteers identified five core components of the Home‐Start program related to positive outcomes for families: providing needs‐oriented care to parents, improving parental empowerment, securing an equal and trusting relationship between parents and volunteers, scheduling frequent (i.e., weekly) home visits, and providing professional support to the volunteer (Lange et al., 2025).

### 1.4 Current study

Since 2019, Home‐Start also provides support to pregnant women and first‐time parents in the Netherlands. However, it has not yet been examined whether the current support services delivered by Home‐Start are equally valuable for this specific target population as they are for families with older children. Examining this is important, as different developmental stages of children may pose distinct challenges for parenting behaviors, and becoming a parent specifically comes with challenges in adapting to the parenting role (Shrestha et al., 2019; Verhoef et al., 2023). Further, the first 1000 days of life are a critical period during which parenting may have an especially strong impact on child development (Britto & Pérez‐Escamilla, 2013; Padgett et al., 2024).

Distinguishing target populations within universal volunteer‐led parenting support programs such as Home‐Start, supports the idea that the most efficient programs components should be delivered to every family given their individual characteristics to optimize program outcomes (Ng & Weisz, 2016). To successfully assist first‐time parents during the transition to parenthood by providing personalized, volunteer‐based parenting support, it is necessary to understand their prospective support needs and their perceived effective elements of the parenting support received (Deave et al., 2008; Warren, 2005). Therefore, we qualitatively examined social (i.e., instrumental, informational, and emotional) needs and perceived effective elements and impact of the Home‐Start program, based on a series of interviews with both enrolled first‐time parents and volunteers. Related to these questions, we also addressed any suggestions for improvements. The results in this study provide valuable insight as regards to the question of what works in volunteer‐led parenting support programs for first‐time parents, and provide insights into opportunities for better tailoring the Home‐Start program to the needs of parents in this decisive life stage.

## 2 METHODS

### 2.1 Participants

Participants in this study included ten first‐time mothers with a first child up to 1.5 years old who were enrolled in the Home‐Start program in the Netherlands for at least six months (*M_age_ *= 33.00*, SD_age_ *= 5.75). Three mothers had a non‐European ethnicity, and four mothers had completed a higher education program (i.e., applied university or university).

In addition, Home‐Start volunteers (*N *= 12) who supported first‐time parents (*M_age_ *= 58.80*, SD_age_ *= 12.40) were included in this study. Five volunteers were paraprofessionals, as they had completed an educational program related to social work or family care. Four volunteers combined their volunteer work with a full‐ or parttime job. The average years of experience as a Home‐Start volunteer varied from a few months to fifteen years (*M *= 4.10 years, *SD* = 4.00). None of the volunteers had a migration background.

### 2.2 Procedure

Parents and volunteers were recruited through convenience sampling by Home‐Start coordinators throughout the Netherlands. Home‐Start coordinators conduct intakes with parents, recruit and train volunteers, match them with families, and supervise their support. Given that all participants had expertise in receiving or providing Home‐Start support, a sufficient degree of data saturation (i.e., the point at which no new information or themes are observed in the data) could be assumed by interviewing six to twelve participants, which was therefore the number we aimed to recruit for both groups (Guest et al., 2006).

Parents were eligible to participate in the study if they: (1) completed—or were nearing completion of—the Home‐Start program; (2) had a first child up to 1.5 years old (i.e., were ‘new’ parents); and (3) possessed sufficient proficiency in Dutch to be able to discuss their experiences with Home‐Start. Volunteers were eligible to participate if they had experience supporting ‘new’ parents, as described above. Parents and volunteers were recruited independently; therefore, they were not specifically paired. All eligible participants were contacted by the coordinators via email or telephone to inform them about the study. If parents or volunteers expressed interest in participating, the coordinator registered them using a secure link that shared their contact information with the researchers.

Although we intended to include both first‐time mothers and fathers, all participating parents in the study were mothers. Participants who agreed to participate received detailed information on research participation and the aims of the study, after which the interview was scheduled with the second and third author. Participants were informed that the interviewers were university graduate students. The interviewers were young adults with a Dutch ethnic background. Interviews took place via an online Teams meeting, or face‐to‐face at home based on participant's preferences. Using an online tool, such as Teams, has proven to be a viable option to collect qualitative data, because it is relatively easy to use, cost‐effective, and secured (Archibald et al., 2019). Before the interview took place, participants signed an online informed consent form by which they actively permitted to record the interview and to use the anonymized data for this study. The average duration of the interviews was 45 min. After the interview, participants received a gift voucher of fifteen euros for their cooperation. This study was approved by the Ethics Committee of the University of Amsterdam, project number FMG‐948. A COREQ (consolidated criteria for reporting qualitative research; Tong et al., 2007) checklist is included in Appendix A. The authors of this manuscript were all students or researchers at a university or a University of Applied Sciences, holding either a bachelor's degree (LdV & RD), PhD (AB & AL) or being a Professor (GO). They are experienced in the field of child and family studies, and AB, AL, and GO had previous experience with qualitative research. All authors are female, expect for GO being male. LdV and RD conducted the interviews. LdV, RD and AB conducted the main analyses. All authors were involved in reading the manuscript and discussing the structure of the subthemes. None of the participants that applied refused to participate after being briefed about the study, and none of the participants dropped out during the study.

### 2.3 The interview

The interview included questions about: (1) background information about the participant (e.g., family composition and work experience) and characteristics of the Home‐Start program (e.g., length); (2) support needs of first‐time parents; (3) effective elements of the Home‐Start program based on the participant's experiences; (4) the volunteer‐parent relationship; (5) specific structure of the home‐visits for first‐time parents; (6) the most important outcomes of the program; (7, only for volunteers) the training, coaching and supervision by Home‐Start coordinators and peer volunteers; and (8) suggestions for aligning the Home‐Start program to the needs of first‐time parents. The interview topics were adapted to align with the perspectives of volunteers and mothers. For example, volunteers were asked: “*What qualities do you consider important when supporting first‐time parents?*”; “*Based on your experience, what should volunteers definitely do or be able to do, and what should they avoid?*”. In contrast, mothers were asked: “*What qualities of the volunteer who supported you did you appreciate most*?”.

### 2.4 Data analysis

The interviews were transcribed and systematically analyzed in ATLAS.ti according to the guidelines of Boeije and Bleijenbergh (2019). In the *open coding stage* of the initial interviews, different quotes were assigned to codes that summarized the core meaning of the statements. In *the axial coding stage*, initial codes were inductively assigned to ‘code groups’. An inductive approach means that categories and themes emerge directly from the data itself rather than being based on pre‐existing theories or hypotheses. In contrast to deductive analyses, where data are analyzed according to predetermined categories or frameworks, inductive analysis allows for openness to new insights during qualitative research (Thomas, 2006). Only during the analysis of the support needs of first‐time parents did theoretical categories serve as a sensitizing framework to guide the initial coding (i.e., instrumental, informational and emotional support), while remaining open to identifying additional support needs emerging from the data. Next, new codes were created, or existing code groups were integrated with corresponding code groups in the *selective coding stage*. A reflexive thematic approach was employed during the coding process, emphasizing the active and interpretative role of the researcher in analyzing the data (Braun & Clarke, 2006). Codes and subthemes were gradually developed through a critical and reflexive engagement with the transcripts. This approach aligns with the principles outlined by Braun and Clarke (2006), in which subjectivity, meaning‐making, and context play a central role. The interviewers were external researchers, and were therefore not involved in the Home‐Start program. This position allowed the researchers to approach the participants with a fresh and independent perspective. At the same time, all researchers brought knowledge and interest from their academic background in child development, which may have influenced their interpretations. To consciously account for this influence, the second and third authors coded all interviews separately for mothers and volunteers, and flagged any discrepancies in the coding procedure. These discrepancies were resolved by discussion with the first author until full consensus was reached. In the end, all authors checked and approved all interview coding themes.

## 3 RESULTS

The following paragraphs present the results from the interviews with first‐time mothers and volunteers in the Home‐Start program. The results were derived from a thematic analysis of the transcripts and are supported by anonymized quotes. Abbreviations were used to refer to mothers, that is, M, or volunteers, that is, V. First, the support needs of first‐time mothers are presented, followed by the perceived effective elements of Home‐Start for first‐time parents. Finally, suggestions are elaborated to better tailor Home‐Start to the target group.

### 3.1 Support needs of first‐time mothers

This section shows an overview of the core support needs of first‐time mothers in the Home‐Start program as described in Table 1.

**TABLE 1:** Support needs of first‐time mothers in the home‐start program.

Themes	Examples
Social support	Expanded social network
Emotional support	Sharing experiences
	Reassurance
Informational support	Baby sleep, nutrition, and health
	Medical questions
	Orientation within society
Instrumental support	Practical support—cleaning, doing laundry, etc.

#### 3.1.1 Social support

Expanding the social network turned out to be a core need of the mothers (*M: ‘I can always call or text the volunteer. It is nice to know that someone is there for you.’*). Home‐Start volunteers also mentioned the importance for mothers to know they do not have to do everything on their own (*V: ‘It is important for mothers to know that there is no shame in asking for help’*).

#### 3.1.2 Emotional support

Mothers appreciated sharing their story with a peer, as they were insecure about their parenting skills (*M: ‘I had no one else to talk to, or to share experiences with about raising my child. No one. That still moves me.’*). Volunteers mentioned the importance of providing emotional support to first‐time parents, specifically to parents in disadvantaged circumstances, for example, refugees who had trauma related symptoms. They sensed the insecurity and fear of parents during the transition to parenthood, and underlined the importance of reassuring that parents are doing a good job (*V: ‘Listening is my main task.’*, or, ‘*When the mother is taking care of her baby, I just stand beside her and comfort her. When she is stressed, I simply touch her on the shoulder to calm her down.'*).

#### 3.1.3 Informational support

Mothers requested information about different topics related to the newborn phase, for example, sleep, nutrition, acid reflux, and health (*M: ‘I have no idea what I am doing, it is nice to discuss those topics with someone.’*). Volunteers noticed that parents also had medical questions (*V: ‘A parent asked me if the red spots on her baby's face were severe. I said that I am not a doctor, but that it looked like a mild skin rash to me, and that there was no reason to panic.’*). Furthermore, volunteers mentioned that they helped migrants find their way within society (*V: ‘For them, life is a rollercoaster as they have to find a job and a home, and learn a new language. Also, they do not know the ins and outs of the way things work around here concerning child and family services.’*).

#### 3.1.4 Instrumental support

Mothers appreciated the help in taking care of the children (*M: ‘I thought, what if something happens, and I would not be able to pick up my baby from daycare? There was no one else who could do that for me. Knowing that I could rely on the volunteer's presence was a huge relief.’*). Volunteers mentioned that parents needed practical support around the house, for example, cleaning, changing diapers, soothing the baby, or getting all the baby necessities together (*V: ‘Parents with young children are terribly busy. It is not the typical Home‐Start approach, but a little help around the house, such as doing the laundry, can be extremely helpful for them.’*).

### 3.2 Perceived effective elements of home‐start for first‐time parents

This section shows an overview of the perceived effective elements of Home‐Start for first‐time parents based on the experiences of first‐time mothers and volunteers as described in Table 2.

**TABLE 2:** Perceived effective elements of home‐start for first‐time parents.

Themes	Examples
Volunteer‐parent supportive bond	Informal relationship
	Availability, e.g., texting
	Equality, acceptance, and genuine interest
Taking time and building trust	Trusting relationship
Positive contact with the coordinator	Reassurance
Volunteers as experienced experts	Advice
	Sharing experiences

#### 3.2.1 Volunteer–parent supportive bond

Both mothers and volunteers appreciated the casual parent‐volunteer relationship (*V: ‘As a volunteer, just being there is enough. Sometimes, a cup of coffee and a brief conversation can cheer a parent up.’*). Furthermore, volunteers mentioned that texting, for example by using WhatsApp, helps building consistent contact. Most of the volunteers indicated that they stay connected with the parents after the Home‐Start program has ended (*V: ‘We still sometimes text via WhatsApp, and I visit the mother once in a while.’*). Further, parents valued a respectful relationship with the volunteer, which was related to equality, acceptance, no judgments, genuine interest, and room for questions. Paraprofessionals navigated a delicate balance between guidance rooted in their professional role and the support they now offered as volunteers, yet they valued the benefits of the volunteer role. For example, one volunteer reported that while the family resisted health care and social service professionals, they were receptive to the support offered by the volunteer (*V: “The family was against all health care and social service workers, but not against me.”*).

#### 3.2.2 Taking time and building trust

Mothers mentioned the importance of taking the time to get acquainted with the volunteer in order to build a trusting relationship, which they considered necessary for letting someone take care of their baby. Volunteers mentioned the importance of an equal relationship (*V: ‘Parents should feel comfortable to share their concerns and discomfort, and therefore, they have to trust you.’*).

#### 3.2.3 Positive contact with the coordinator

Mothers felt reassured by the Home‐Start coordinators in asking for, and receiving care (*M: ‘She reassured me that it was a good thing to ask for help, because I had just moved to another city, and I was recovering from a burnout. She felt like a mother figure to me.’*).

#### 3.2.4 Volunteers as experienced experts

The core program component that mothers evaluated as effective was the way volunteers functioned as experience experts. They highly valued the volunteers’ advice and the way they shared their own experiences with the parents (*M: ‘We had both lost a baby in the past, so she knew how to cope with grief and loss. She shared her story with me.’*).

### 3.3 Perceived outcomes of home‐start

This section shows an overview of the perceived outcomes of Home‐Start for first‐time parents based on the experiences of first‐time mothers and volunteers as described in Table 3.

**TABLE 3:** Perceived outcomes of home‐start for first‐time parents.

Themes	Examples
Social support	Friendship
Parental confidence and positive parent‐child interactions	Parental competence

#### 3.3.1 Social support

Mothers mentioned that the most important outcome of the Home‐Start program was the social support they received from the volunteer (*M: ‘Despite the fact that she is older than me, she turned into a dear friend*.’).

#### 3.3.2 Parental confidence and positive parent–child interactions

Furthermore, mothers said that Home‐Start strengthened their confidence and parent‐child interactions (*M: ‘I am actually enjoying spending time alone with my baby now at home instead of feeling stressed.’*, or, *M: ‘Home‐Start helps building a positive home environment, including a positive mindset which is very important for first‐time parents.’*). According to the volunteers, strengthening parental confidence and competence should be the main aim of Home‐Start for first‐time parents (*V: ‘At the end of the program, it is important that parents feel competent enough to take care of their baby, and that they know that there is no shame in asking for help.’*).

### 3.4 Home‐start for first‐time parents: further suggestions

This section shows an overview of further suggestions to better tailor Home‐Start to the needs of first‐time parents as described in Table 4.

**TABLE 4:** Suggestions to better tailor home‐start to the needs of first‐time parents.

Themes	Examples
Quicker matching process	Parent‐volunteer match during pregnancy
Raise awareness	Gain visibility
Diversify the campaign to accurately target different parent groups	Break down barriers to ask for help
Training and supervision of volunteers	Additional/optional module on supporting parents during the transition to parenthood
Structure and boundaries	Defined structure of the home visits

#### 3.4.1 Quicker matching process

Mothers indicated that the process from application to the actual matching and start of the program sometimes took too long, which affected the relevance of the support in the transition to parenthood (*M: ‘I am sure that the help would have been more valuable when we had met earlier.’)*. Further, mothers said that they needed the Home‐Start support from the beginning of the post‐partum phase (*M: ‘I needed support directly after giving birth, it would have been very helpful when the volunteer could have started then’; M: ‘The first weeks with a newborn are very intensive, it helps when you have already met the volunteer during the third trimester.’*).

#### 3.4.2 Raise awareness

Mothers suggested to gain visibility of the Home‐Start program via general practitioners, midwives, and postnatal care services (*M: ‘I literally cried when someone told me that Home‐Start existed. I genuinely wish someone would have told me about the program sooner.’*).

#### 3.4.3 Diversify the campaign to accurately target different parent groups

Highly educated mothers did not directly identify themselves with the examples of families at the Home‐Start website or in the Home‐Start flyers (*M: ‘It should be clear that Home‐Start is for everyone, despite income, background, or level of education. Personally, I experienced a barrier signing up for the program.’*).

#### 3.4.4 Training and supervision of volunteers

Volunteers were divided on whether an additional module on supporting parents during the transition to parenthood should be included in the Home‐Start training (*V: ‘I have never signed up for a pregnancy or baby course, but I doubt if that would be necessary. In my opinion, volunteers that are parents themselves do not need additional training.’*, or ‘*V: Sometimes parents ask me questions about medical or psychological conditions. I had children thirty years ago, so my knowledge about those topics is probably outdated.’*). Furthermore, volunteers indicated that it would be helpful to have a document outlining local infant health care facilities and organizations for referral purposes.

#### 3.4.5 Structure and boundaries

Some mothers felt that the home visits could have benefited from a more defined structure (*M: ‘The volunteer said that I had to suggest the aims of the visits. I found that rather difficult because I am not used to ask for help. It would have been helpful when the volunteer would have listed some specific Home‐Start topics that she could help me with.’*). On the other hand, volunteers mentioned that they had trouble guarding their boundaries (*V: ‘It is hard to find a balance between being there for the mother, and operating like a free babysitter.’*).

## 4 DISCUSSION

The aims of this qualitative study were to examine the needs of first‐time parents who were enrolled in the Home‐Start program, and to examine their—and their volunteers’—perspectives on what are effective program components and overall impact of Home‐Start. The findings indicate that Home‐Start is a valuable volunteer‐led parenting support program for first‐time mothers.

### 4.1 Support needs of first‐time mothers in the home‐start program

First‐time mothers that were enrolled in the Home‐Start program needed an expansion of their social network for instrumental support (e.g., practical help with daily tasks and baby care), informational support (e.g., advice on infant health, sleep, and nutrition), and emotional support (e.g., a sympathetic ear) (Cohen, 2004). In previous studies, mothers were found to experience mixed emotions following the birth of their newborn, and they needed that health care providers acknowledged these emotions and reassured them (Schobinger et al., 2022). In other words, “*they just wanted that someone would ask them how they felt*” (Mcleish et al., 2020; Schobinger et al., 2022). Volunteer‐led parenting support during the postpartum period can address these needs by filling the gap left by formal health care providers, such as midwives, once they are no longer involved.

### 4.2 Core components of the home‐start for first‐time parents

The results of this study indicated that several integrated principles (e.g., mutual trust and providing a listening ear) of the Home‐Start program are just as important to first‐time parents as they are for parents with older children (Lange et al., 2025).

Both mothers and volunteers valued the informal parent‐volunteer relationship as a core component of the Home‐Start program (Dowling, 2019; Gross Manos et al., 2023). More specifically, mothers appreciated a relationship based on trust and equality in line with the view that a hierarchical relationship is likely to alienate clients in social services (Lee, 2003). This informal relationship corresponds to the proposed *befriending* peer support model of the Home‐Start program, which is defined by frequent visits, spontaneous interaction during visits, sharing personal contact details, and a flexible ending (McLeish & Redshaw, 2015). On the other hand, first‐time mothers indicated that they might prefer more structured visits, which can be defined by using facilitators or activities and setting and reviewing goals as described in the *mentoring* peer support model (McLeish & Redshaw, 2015). However, the perceived ‘sitting back attitude’ of Home‐Start volunteers may actually relate to the complicated skill of empowering clients (Schaefer, 2016).

A second program component that mothers evaluated as effective was the way volunteers functioned as experts who experienced similar life transitions. These findings are in line with findings reported in the USA, where one in three of postpartum women wanted to gain more self‐confidence, and more baby care information in the first seven weeks following childbirth, whether or not they had antenatal education (Hanna et al., 2002; Moran et al., 1997). Home‐Start volunteers aim to address this gap by enhancing maternal sense of competence through practical assistance and emotional support (Deković et al., 2010).

### 4.3 Perceived outcomes of home‐start

Accordingly, important outcomes for the participants of this study included more social support, improved parental confidence, and increased positive parent‐child interactions. These results demonstrate that support provided through the Home‐Start program can assist new parents after childbirth by fostering bonding with their baby and enhancing their confidence and sense of security (Schobinger et al., 2022). These findings align with the principles of self‐efficacy theory (Bandura, 1997), suggesting that parents who feel more confident in their parenting abilities are more likely to engage in positive parenting behaviors.

### 4.4 Home‐Start for first‐time parents: further suggestions

The interviewed mothers and volunteers offered several suggestions to better align the program's structure to parents in the transition to parenthood. First, participants suggested to facilitate the matching between parents and volunteers during pregnancy. This allows an immediate start of the program after birth to enhance the well‐being of parents during the physically and emotionally demanding phase of the transition to parenthood (Halford et al., 2010; Pinquart & Teubert, 2010).

Second, participants suggested to focus on dispelling prejudices about volunteer‐led services, and on increasing awareness of Home‐Start's support services. Aligning Home‐Start's promotional material with norms, values, and perspectives of, for example, theoretically educated parents may contribute to recognition and bridging the taboo on asking for help for these parents (Knipscheer, 2020).

Third, in addition to the aims of Home‐Start, instrumental support (e.g., household assistance and baby care) appears to be a key component in promoting the physical and emotional well‐being of first‐time parents (Negron et al., 2013). To offer appropriate postpartum support, volunteers need sufficient knowledge about infant care (e.g., feeding, sleep, developmental stages, and health care), and the local network of social and child and family services for referral (Leger & Letourneau, 2014).

Last, introducing a specific module in the Home‐Start training aimed at providing structured support for first‐time parents may provide volunteers with the tools they need to address parent's needs during the postpartum phase. The flexible, needs‐led approach by Home‐Start volunteers may cause struggles in conveying exactly what they offer, and cause uncertainty among first‐time parents regarding what they can ask for or expect (McLeish & Redshaw, 2015).

### 4.5 Study strengths and limitations and further research

This study explores the perceived value and key components of a community‐based, volunteer‐led parenting support program during the transition to parenthood, drawing on the experiences of both first‐time mothers and volunteers. A qualitative approach enabled an in‐depth exploration of the underlying values, beliefs, and assumptions held by both groups. The use of broad and open‐ended questions allowed participants to discuss the issues most meaningful to them, potentially yielding richer and more nuanced insights than would be captured through survey methods (Choy, 2014). To ensure trustworthiness, the researchers engaged in reflexivity throughout the coding process, used independent double coding followed by consensus meetings to reduce bias, and supported themes in the result section with illustrative quotes from participants.

Although the present study included a sample that provided sufficient data saturation (Boeije & Bleijenburgh, 2019; Guest et al., 2006), it is appropriate to recognize a potential selection bias due to the direct recruitment of participants by Home‐Start coordinators (Maruyama & Ryan, 2014). Volunteers and parents who were willing to participate in this study may have been intrinsically motivated to share their experiences with the program, which could potentially have led to a one‐sided evaluation. However, by offering participants compensation for their time, participation from a more diverse range of individuals was encouraged, including those who might not have taken part otherwise.

In addition, this study only involved mothers, and this may limit the study's generalizability as preferably also fathers should have reported on needs, outcomes, and suggestions. In general, there is still little attention given to the support needs of fathers, even though there is an increasing expectation of active participation from fathers in parenting (Travecchio, 2015). Including fathers in studies on parenting support programs is often challenging, as observed in several previous studies on the Home‐Start program, where data was collected only from mothers (Asscher et al., 2008; Hermanns et al., 2013; Smallegange et al., 2018).

Last, this explorative study on the value of Home‐Start offers valuable insights into potential effective components (e.g., providing emotional‐, informational‐, practical‐, and social support) of volunteer‐led parenting support programs for first‐time parents. Preferably, the effectiveness of those moderators should be examined in further longitudinal research. Such studies would allow for tracking outcomes over time, identifying which components have lasting effects, and determining how these components interact to influence family well‐being and child development.

### 4.6 Study implications

Family dysfunction that might be managed through informal support networks often end up receiving unnecessary, expensive professional intervention. This places additional strain on youth care professionals, contributes to growing waiting lists, and ultimately disadvantages the families who genuinely require specialized care, forcing them to wait long for the help they need (Overbeek, 2024). Given that infant's interactions with their parents play a vital role in shaping biological, cognitive, emotional, and social development, and can have lasting effects on their future social relationships and psychological well‐being, early prevention is critical (Kim et al., 2013). The results of this study indicate that a volunteer‐based home visiting program, such as Home‐Start, can provide valuable support to first‐time parents by strengthening their confidence and promoting positive parenting behaviors. In the long term, such early support may help to prevent more serious problems from developing in families and children.

## 5 CONCLUSION

This study offers important insights into the specific emotional, informational, and instrumental support needs of first‐time parents participating in Home‐Start, a globally recognized volunteer‐led parenting support program. The results highlight the role of volunteer‐led home visiting support in strengthening parent‐child interactions and enhancing parental confidence throughout early childhood development. Additionally, the study underscores the value of an informal, equal relationship between volunteers and parents, as well as the significance of volunteers acting as experience‐based experts. By recommending the development of a structured training module for volunteers grounded in the latest evidence on infant and postpartum care, this study provides useful guidance that could enhance program implementation. Moreover, it is advised to initiate early matching of parents with volunteers during pregnancy, allowing ample time to build trust. Furthermore, it is essential to actively promote that asking for help is encouraged for all first‐time parents who feel they are facing challenges alone. Ultimately, these findings contribute to the field by informing more targeted and responsive early parenting—informal—support interventions that can strengthen family resilience.

## CONFLICT OF INTEREST STATEMENT

The authors report there are no competing interests to declare.

## Supporting information



Supporting‐Information

## Data Availability

The data that support the findings of this study are available from the corresponding author, A. M. E. Bijlsma, upon request.
