# Supplementary material for: Tailoring the Home‐Start program to the needs of first‐time parents: A qualitative study
Source: Infant Ment Health J. 2025 Sep 30;47(1):e70048. doi: 10.1002/imhj.70048 (PMC12719908; doi:10.1002/imhj.70048)
Supplement: Supplementary file 1 — Supporting‐Information [file IMHJ-47-0-s001.docx]

**Appendix 1**

**COREQ (COnsolidated criteria for REporting Qualitative research) Checklist**

| **Topic** | **Item no.** | **Guide questions/description** | **Reported on page no.** | **Text in manuscript** |
| --- | --- | --- | --- | --- |
| **Domain 1: research team and reflexivity** | | | |  |
| *Personal characteristics* | | | |  |
| Interviewer/  facilitator | 1 | Which author/s conducted the interview or focus group? | 11 | LdV and RD conducted the interviews. |
| Credentials | 2 | What were the researcher’s credentials? e.g., PhD, MD | 11 | The authors of this manuscript were all students or researchers at a University or a University of Applied Sciences, holding either a bachelor’s degree (LdV & RD), PhD (AB & AL) or being a Professor (GO). |
| Occupation | 3 | What was their occupation at the time of the study? | 11 | See item 2 |
| Gender | 4 | Was the researcher male or female? | 11 | All authors are female, expect for GO being male. |
| Experience and training | 5 | What experience or training did the researcher have? | 11 | They are experienced in the field of child and family studies and volunteer-based support programs and AB, AL, and GO had previous experience with qualitative research. |
| *Relationship with participants* | | | |  |
| Relationship established | 6 | Was a relationship established prior to study commencement? | 12 | The interviewers were external researchers, and were therefore not involved in the Home-Start program. |
| Participant knowledge of the interviewer | 7 | What did the participants know about the researcher? e.g., personal goals, reasons for doing the research | 10 | Participants who agreed to participate received detailed information on research participation and the aims of the study, after which the interview was scheduled with the second and third author. |
| Interviewer characteristics | 8 | What characteristics were reported about the interviewer/facilitator? e.g., bias, assumptions, reasons, and interests in the research topic | 10 | Participants were informed that the interviewers were university graduate students. |
| **Domain 2: study design** | | | |  |
| *Theoretical framework* | | | |  |
| Methodological orientation and theory | 9 | What methodological orientation was stated to underpin the study? e.g., grounded theory, discourse analysis, ethnography, phenomenology, content analysis | 12 | The interviews were transcribed and thereafter systematically analyzed in ATLAS.ti according to the guidelines of Boeije and Bleijenburg (2019). |
| *Participant selection* | | | |  |
| Sampling | 10 | How were participants selected? e.g., purposive, convenience, consecutive, snowball | 10 | Parents and volunteers were recruited through convenience sampling by Home-Start coordinators throughout the Netherlands. |
| Method of approach | 11 | How were participants approached? e.g., face-to-face, telephone, mail, email | 10 | See item 10 |
| Sample size | 12 | How many participants were in the study? | 9/10 | Participants in this study included ten first-time mothers with a first child up to 1.5 years old who were enrolled in the Home-Start program in the Netherlands for at least six months; In addition, Home-Start volunteers (N = 12) who supported first-time parents were included in this study. |
| Non-participation | 13 | How many people refused to participate or dropped out? Reasons? | 11 | None of the participants that applied refused to participate after being briefed about the study, and none of the participants dropped out during the study. |
